# Krill oil inhibited adipogenic differentiation by inducing the nuclear Nrf2 expression and the AMPK activity

**DOI:** 10.1002/fsn3.3576

**Published:** 2023-07-21

**Authors:** Hyun Jeong Lee, Ji Won Seo, Yoon Seok Chun, Jongkyu Kim, Tae‐Gyu Lim, Soon‐Mi Shim

**Affiliations:** ^1^ Department of Food Science and Biotechnology Sejong University Seoul Korea; ^2^ Department of Agricultural Biotechnology and Research Institute of Agriculture and Life Sciences Seoul National University Seoul Korea; ^3^ Ari B&C Co Gyeonggi‐do Korea

**Keywords:** adipogenesis, adipogenic gene, krill oil, phosphatidylcholine

## Abstract

The current study investigated the antiadipogenic mechanism of krill oil from the 3T3‐L1 adipocytes. The krill oil adhered to the criteria as a food standard by showing 50.8% of the total phospholipid, 5.27% myristic acid, and 1.63% linoleic acid. The lipid accumulation that was measured in the 3T3‐L1 cells using oil red O staining was reduced up to 54% by the krill oil. The krill oil treatment reduced the adipogenic transcription factors by downregulating the sterol regulatory element binding protein 1 (SREBP1) and acetyl‐CoA carboxylase (ACC), phospho‐ACC, and AMP‐activated protein kinase (AMPK) phosphorylation. The current study confirmed that the krill oil inhibited adipogenesis by downregulating SREBP1 and ACC via the upregulation of the AMPK and nuclear factors E2‐related factor 2 (Nrf2) signaling pathway in the 3T3‐L1 adipocytes. These findings suggest that krill oil is a good source of phospholipid and phosphatidylcholine, which could be a potential natural antiobesity ingredient by inhibiting adipogenesis.

## Graphical abstract

The krill oil containing myristic acid (5.3%), linoleic acid (1.6%), and phospholipids (50.8%) inhibited lipid accumulation by 54% at 400 μg/mL. Standardized krill oil reduced SREBP1, ACC, and p‐ACC expression by activating Nrf 2 nuclear expression and inhibiting adipogenesis.

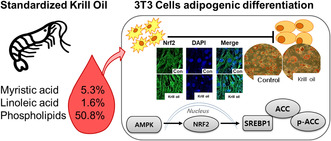

## 1 INTRODUCTION

Obesity is a global epidemic, even though the members of the World Health Organization member stated that a voluntary goal is to prevent an increase in obesity by 2025 (Ng et al., 2014; Williams et al., 2015). It is predicted that 20% of the worldwide population will be influenced by obesity, and 38% of the population will be overweight by 2030 (Al‐Ghamdi et al., 2018). Being overweight and obese, which means having excess body fat, is a serious disease that can cause or exacerbate various metabolic disorders, which include dyslipidemia, cardiovascular disease, and diabetes (Chu et al., 2018; Di Cesare et al., 2019). Thiazolidinediones, hypolipidemic agents, and antihyperglycemic agents for the treatment of obesity are known to have side effects, such as hepatotoxicity (Nanjan et al., 2018). According to a previous finding, about 54 natural substances have shown their antiobesity potential, but the mechanisms of antiobesity are not enough to understand the therapeutic potential in the scientific field (Karri et al., 2019).

Krill oil is known to be a supplement for reducing metabolic syndrome by lowering the triglycerides, hip ratio, visceral fat, and blood glucose level (Berge et al., 2013; Di Marzo et al., 2010). It is unlike most marine oils, because it is a unique source of docosahexaenoic acid (DHA) and eicosapentaenoic acid (EPA), which are known to provide health benefits in regard to increasing high‐density lipoprotein (HDL) cholesterol and prevent heart disease and inflammation (Araujo et al., 2014; Schuchardt et al., 2011). Krill oil that contains n‐3 polyunsaturated fatty acids (n‐3 PUFA), which is one of the phospholipids, has been reported to promote lipid metabolism (Vigerust et al., 2013), and it reduces triglycerides, glucose, and low‐density lipoprotein (LDL) levels in order to control hyperlipidemia (Bunea et al., 2004). Several studies also confirmed the properties of phospholipids especially phosphatidylcholine in krill oil (Le Grandois et al., 2009; Winther et al., 2011). These beneficial effects on human health have increased the consumption and the research interests of krill oil (Araujo et al., 2014; Le Grandois et al., 2009).

Nuclear factor E2‐related factor 2 (Nrf2) is known as the master regulator which controls the transcriptional activation of genes involved in antioxidant biosynthesis, antioxidation, and metabolic shift (Kasai et al., 2020). The Nrf2, which is a transcription factor that is known as the key antioxidant regulators, has a critical role in the adipose tissue differentiation (Kim et al., 2018). A previous study showed that activation of Nrf2 is protected from obesity (Kasai et al., 2020). The stabilized Nrf2 is translocated into the nucleus, which then activates its target genes, such as the AMP‐activated protein kinase (AMPK) phosphorylation (Chang et al., 2018; Kobayashi & Yamamoto, 2005). The AMPK, which is a central regulator of inflammation and cellular energy homeostasis, modulates fatty acid biosynthesis through interactions, and it directly phosphorylates with the sterol regulatory element binding protein 1 (SREBP1) and acetyl‐CoA carboxylase (ACC) (Lee et al., 2018; Li et al., 2011). However, studies that determine the mode of action in regard to the antiobesity effect of krill oil are lacking. Thus, the protein genes that are concerned with the lipid metabolism should be further studied. The aims of the current study were to investigate the effect of the krill oil on the lipid accumulation by measuring the adipogenic factors, which included phospho‐AMPK (p‐AMPK), ACC, phospho‐ACC (p‐ACC), and SREBP1 in the protein level and to elucidate the mode of action that is related the transactivation of Nrf2.

## 2 MATERIALS AND METHODS

### 2.1 Chemicals and reagents

The newborn bovine calf serum (BCS), Dulbecco's modified eagle's medium (DMEM), and fetal bovine serum (FBS) were bought from Thomas Scientific Inc. The Dulbecco's phosphate buffer saline (DPBS) and the trypsin solution were purchased from Corning Inc. The penicillin–streptomycin was obtained from Biotechnic Research, Inc. The isopropyl alcohol, n‐hexane, chloroform, HPLC water, triethylamine, and methanol with the HPLC grade were purchased from Sigma‐Aldrich. The standards for the phosphatidylcholine were purchased from Larodan AB (CAS 97281–47‐5; Larodan). The boron trifluoride–methanol solution (BF3‐MeOH), sodium hydroxide (NaOH), isooctane, acetic acid, sodium sulfate anhydrous, dimethyl sulfoxide (DMSO), 3‐(4‐5‐dimethylthiazol‐2‐yl)‐2.5‐dyphenyltetrazolium bromide (MTT), orlistat, insulin, 3‐isobutyl‐1‐methylxanthine (IBMX), and the dexamethasone were purchased from Sigma‐Aldrich. The phospho‐AMPKα was purchased from Cell Signaling Technology. The ACC mouse mAb, SREBP1, and phospho‐ACC were purchased from Santa Cruz Biotechnology. The krill oil from Antarctic krill (*Euphausia superba*) was supplied by Aribio H&B, which was stored at 4°C without exposure to light until the end of the experiment.

### 2.2 Sample preparations

The krill oil (Suoperba™ Boost) that was produced by Aker Biomarine was purchased from SC Science. The Antarctic krill meal was heated using steam, and it was then centrifuged. It was dried and then extracted using ethanol. The supernatant was filtered in order to remove any particles, and it was purified. The final batch was filtered, and it was then packed in Food and Drug Administration (FDA)‐approved food contact materials. It was then blanketed with a nitrogen headspace and sealed. The batch was stored at 4°C without exposure to light until the end of the experiment. The krill oil contained 56% (*wt*/*wt*) of the total phospholipids according to the certificate of analysis (COA) that was provided by SC Science.

### 2.3 Analysis of the phospholipids and fatty acids from the krill oil

The amount of phospholipids from the krill oil was measured using the method from the Ministry of Food and Drug Safety (MFDS, 2021). The krill oil was diluted with n‐hexane: isopropanol (8:2, *v/v*) prior to the high‐performance liquid chromatography (HPLC)‐evaporative light scanning detector (ELSD) analysis. The identification and quantification of the phospholipids were conducted using an HPLC‐ ELSD system with a Lichrospher 100 Diol column (5 μm, 250 × 4 mm, Merck KGaA) at 55°C. The mobile phase A included n‐hexane: isopropanol: acetic acid: triethylamine = 81.42:17:1.5:0.08 (*v/v/v/v*), and the mobile phase B included n‐hexane: isopropanol: acetic acid: triethylamine = 84.42:14:1.5:0.08 (*v/v/v/v*). The gradient was composed: 0–5 min, 5%–20% B; 5–8.5 min, 20%–40% B; 8.5–15 min, 40%–100% B; 15–17.5 min, 100%–5% B; and 17.5–34 min, 5% B. The quantitative data were obtained in conditions with a flow rate of 1.0 mL/min, which was conducted for 34 min with an injection volume of 20 μL. The content of the phospholipids was calculated as follows:

$$\begin{array}{c} \text{Phospholipid content}\;\left(\frac{\mathrm{wt}}{\mathrm{wt}}\%\right)=\text{phosphatidylcholine}\;\left(\mathrm{wt}/\mathrm{wt}\%\right)\\ +\text{lysophosphatidylcholine}\;\left(\mathrm{wt}/\mathrm{wt}\%\right)+\text{phosphatidylethanolamine}\;\left(\mathrm{wt}/\mathrm{wt}\%\right)\\ +N-\text{acyl}-\text{phosphatidylethanolamine}\;\left(\mathrm{wt}/\mathrm{wt}\%\right) \end{array}$$

The fatty acids from the krill oil were quantified according to the analytical standard that was issued by MFDS (MFDS, 2021). An aliquot amount of krill oil (25 mg) was mixed with a 0.5 N NaOH‐MeOH solution that was 15 mL and heated using a heating block for 5 min at 100°C. After cooling the mixture to room temperature, 2 mL of the 14% (*w/v*) BF3‐MeOH was added and reacted at 100°C for 30 min. The reaction product was again cooled down at 30–40°C and vortexed vigorously for 30 s after 1 mL of isooctane was added. The 5‐mL sodium chloride solution was then vigorously mixed and cooled down to room temperature in order to form a separate organic layer from the aqueous layer. The organic phase that contained the isooctane was dehydrated by sodium sulfate anhydrous and analyzed. Identification of the myristic acid and the linoleic acid was conducted using a gas chromatograph (Agilent Technologies Co. Ltd) with a flame ionization detector (GC/FID), which included an SP‐2560 column capillary GC column (100 m × 0.25 mm, 0.20 μm). The column was retained at 100°C for 4 min, and it was then raised to 240°C at a rate of 3°C/min. The temperature was then maintained at 240°C for 15 min. The temperature of the injector was set at 225°C, and the detector's temperature was set at 285°C. Helium was used at a 0.75 mL/min flow rate as a carrier gas, which included a split ratio of 200:1. Each sample was analyzed using three replicates, and they were quantified against the retention times to the standard solution.

### 2.4 Cell culture and differentiation

The 3T3‐L1 cells were cultivated in a DMEM that was supplemented with 10% (*v/v*) BCS and 1% (*v/v*) penicillin/streptomycin with 5% CO_2_ and 95% air at 37°C. For the cell differentiation into mature adipocytes, the 3T3‐L1 cells were seeded in six‐well culture plates at 1.0 × 10^5^ cells/well, which were cultured to 100% confluency. After the 100% confluence, which was day 0, the adipocyte differentiation was induced in a DMEM, which included 10% FBS with a 0.5 mM IBMX, 10 μg/mL of insulin, and 1 μM of dexamethasone. The cells were incubated for 3 days, and they were then cultured to the changed medium with DMEM that contained 10% (*v/v*) FBS and insulin (10 μg/mL). The cells were then replaced with a new medium, which included a DMEM with 10% FBS and 10 μg/mL insulin until the end of the differentiation (day 10), every other day. Each sample concentration including 0, 50, 100, 200, and 400 μL/mL was added to the cells when the medium was replaced.

### 2.5 Cell viability assay

The cell viability was performed using an MTT assay that included a 3T3‐L1 cell (Chin‐Lin Hsu and Gow‐Chin Yen., 2007). The 3T3‐L1 preadipocytes were seeded at a density of 1 × 10^4^ cells/well into 96‐well microtiter plates. The culture medium was removed after 24 h, and treated with various krill oil concentrations of 0–6400 μg/mL for 24 h. The culture medium was removed after 24 h, the MTT solution (0.5 mg/mL) was added to each well (100 μL/well), and the plate was incubated for 4 h at 37°C. The absorbance of the dissolved formazan crystals into the DMSO (100 μL/well) was measured using a microplate reader (Thermo Scientific Varioskan Flash) at 570 nm. The cell viability percentage (%) was calculated as the % of the cell growth compared to the control group, which is the cells that were not treated with krill oil.

### 2.6 Lipid accumulation by oil red O staining

Lipid accumulation was achieved by oil red O staining in mature adipocytes. The differentiated mature adipocytes (10 days) were rinsed with DPBS three times and fixed with 3.7% (*v/v*) formaldehyde (Sigma‐Aldrich) for 15 min. After the formaldehyde was removed, the cells were rinsed with DPBS, and they were then stained with the oil red O solution for 20 min. The stained cells were observed after being rinsed several times using a microscope (CKX41SF; OLYMPUS), and the lipid droplet was imaged at ×200 magnification using a digital camera (SONY DSLR‐A500). In order to quantify the lipid accumulation, the stained cells were further extracted using 100% isopropanol, and they were then measured at 510 nm using a microplate reader (Varioskan Flash, Thermo Fisher Scientific).

### 2.7 Western blot analysis

The protein lysates of the differentiated 3T3‐L1 cells were collected using a RIPA lysis buffer (Bio‐Rad) that was supplemented with a protease/phosphatase inhibitor cocktail (Cell Signaling Technology). The nuclear extracts were prepared using the Nuclear Extraction Kit (Abcam) according to the instructions from manufacturers in order to evaluate the Nrf2. The protein concentration was determined using a bicinchoninic acid (BCA) analysis (Thermo Scientific Varioskan Flash). The protein was separated using SDS–polyacrylamide gel (SDS‐PAGE) electrophoresis. The protein was transferred into an Immobilon‐P membrane, and the protein was blocked by 5% fat‐free milk for 1 h at room temperature. The membranes were then incubated for 16 h at 4°C with a specific primary antibody, which was then incubated with the corresponding secondary antibodies (1:10,000) for 1 h at room temperature. Finally, the protein band was visualized using an image analysis system (WSE‐6200; LuminoGraph Chemidoc, ATTO) that included a Super Signal™ West Femto Maximum Sensitivity Substrate (Thermo Scientific). The intensity of each western blot band was quantified using the ImageJ (NIH) software.

### 2.8 Immunofluorescence

The cells that were grown on the krill oil‐pretreated solution were fixed with 4% (*v/v*) paraformaldehyde for 15 min, and they were then washed with PBS three times. Next, the cells were treated with 5% bovine serum albumin for 1 h at room temperature for the blocking, which was then incubated with the Nrf2 antibodies (1:250; Abcam) overnight at 4°C. The cells were incubated after that with a fluorescently conjugated anti‐mouse secondary antibody, which was Alexa fluor 488 (1:1000; Abcam). The cellular nuclei were then stained with DAPI (Abcam). Finally, the cells were treated with a mounting medium (ProLong Gold; Thermo Fisher Scientific) for the fluorescence. The stained cellular nuclei were imaged using an inverted confocal laser scanning microscope (ZEISS).

### 2.9 Statistical analysis

All the pieces of data are indicated as means ± standard deviation (SD) for the three unattached experiments. The analysis of variance was applied in order to measure the significance of the dissimilarity of the means between the treated sample and the control. The differences between the mean values of the multiple groups were analyzed using a one‐way analysis of variance. The values of *p* < .05, *p* < .01, and *p* < .001 were regarded to be statistically significant.

## 3 RESULTS AND DISCUSSION

### 3.1 Standardization of the krill oil

The composition of the phospholipids and their content (*wt*/*wt*%) from the three lots of krill oil is presented in Table 1. As a quality index of krill oil, phosphatidylcholine (PC), lysophosphatidylcholine (LPC), phosphatidylethanolamine (PE), and N‐acyl‐Phosphatidylethanolamine (NAPE) were identified. It was quantified as 44.32 ± 0.49, 3.69 ± 0.11, 2.17 ± 0.09, and 0.62 ± 0.02 *wt*/*wt*%, respectively (Table 1). This was equal to 50.80 ± 0.35 *wt*/*wt*% of the total phospholipids from the krill oil, which indicated less than 20% of the variation from the COA (56 *wt*/*wt*%). A relatively higher portion of PC was observed compared to the LPC, PE, and NAPE (Xie et al., 2017), which was similar to our finding. According to the krill oil monograph of the United States Pharmacopeia (USP), which defined that 60%–96% of the total phospholipids is phosphatidylcholine (United States Pharmacopeia, 2017), the krill oil that is used in the current study was acceptable.

**TABLE 1:** The amount of phospholipids and phosphatidylcholine from the krill oil.

Name	Content (wt/wt%)
Phosphatidylcholine	44.32 ± 0.49^a^
Lysophosphatidylcholine	3.69 ± 0.11
Phosphatidylethanolamine	2.17 ± 0.09
N‐acyl‐Phosphatidylethanolamine	0.62 ± 0.02
Total Phospholipid	50.8 ± 0.35^a^

^a^ Data are presented as mean ± SD (*n* = 3).

The composition of the fatty acids in the krill oil is shown in Table 2. The 11 fatty acids, which included myristic acid, palmitic acid, *cis*‐palmitoleic acid, elaidic acid, oleic acid, linoleic acid, eicosenoic acid, erucic acid, linolenic acid, α‐linolenic acid, eicosapentaenoic acid (EPA), and docosahexaenoic acid (DHA) were identified. The EPA, palmitic acid, and DHA were the most abundant in the krill oil. A previous study reported that the content of EPA, palmitic acid, and DHA acid in the total fatty acid was the most abundant, which showed 19.92, 23.46, and 23.47%, respectively (Ahn et al., 2018). The DHA and EPA are metabolically synthesized from linolenic acids, and linoleic acids are widely known to be functional fatty acids in order to support brain functions and cardiovascular health, and reduce triacylglycerol (Kopecky et al., 2009). The content of myristic acid was identified to be 5.27 ± 0.54%, and the linoleic acid was identified to be 1.63 ± 0.15%. The recent Ministry of Food and Drug Safety (MFDS) proposed criteria, which stated that krill oil should not contain less than 3% of linoleic acid as well as 5%–13% of myristic acid (2021). The krill oil that was used in the current study is acceptable for food ingredients considering the criteria for krill oil that were defined by the MFDS.

**TABLE 2:** Composition of the fatty acids from the krill oil.

Fatty acid	Shorthand notation	Content (wt/wt%)
Saturated fatty acids		
Myristic acid	C14:0	5.27 ± 0.54^a^
Palmitic acid	C16:0	14.66 ± 1.01
Monounsaturated fatty acids		
*cis*‐Palmitoleic acid	C16:1 n‐7, cis	3.48 ± 0.22
Elaidic acid	C18:1n‐9, trans	0.09 ± 0.02
Oleic acid	C18:1n‐9, cis	10.02 ± 0.47
Eicosenoic acid	20:1 n‐9	0.34 ± 0.03
Erucic acid	22:1 n‐9	0.51 ± 0.01
Polyunsaturated fatty acids		
Linoleic acid	18:2 n‐6, cis	1.63 ± 0.15
*α*‐Linolenic acid	18:3 n‐3	1.42 ± 0.33
Eicosapentaenoic acid (EPA)	20:5 n‐3	16.49 ± 1.97
Docosahexaenoic acid (DHA)	22:6 n‐3	9.61 ± 1.68

^a^ Data are presented as mean ± SD (*n* = 3).

### 3.2 Effect of krill oil on cell viability in the 3T3‐L1 cell

**FIGURE 1:**
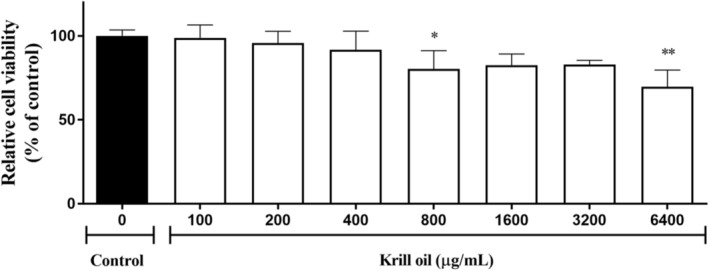
The cell viability of the krill oil doses according to the concentration (0–6400 μg/mL) in the 3T3‐L1 cells.

The 3T3‐L1 cells were treated with various concentrations of krill oil for 24 h. The effect of the krill oil on cell viability was assessed by the 3T3‐L1 cells by using an MTT assay. The cell viability was above 80% in all doses from 100 to 3200 μg/mL except for the 6400 μg/mL concentration. This indicated that the viability ranged from 98.8% to 82.4% of the control group, which is shown in Figure 1. The results imply that the krill oil did not have an effect on the cell cytotoxicity at the dose level (100–3200 μg/mL). Similar to our finding, the previous reports found that a krill oil treatment up to 200 μg/mL concentration in HepG2 not only showed cell viability which was above 80% but also remarkably reduced the lipid droplet size in the HepG2 cells (Lee et al., 2015). Especially, the cytotoxicity appeared to be completely unaffected under 400 μg/mL of rGL in the 3t3‐L1 cells. The following experiments were performed at noncytotoxic concentrations of 50, 100, 200, and 400 μg/mL based on this result.

### 3.3 Effect of the krill oil on the lipid accumulation in the 3T3‐L1 adipocyte

**FIGURE 2:**
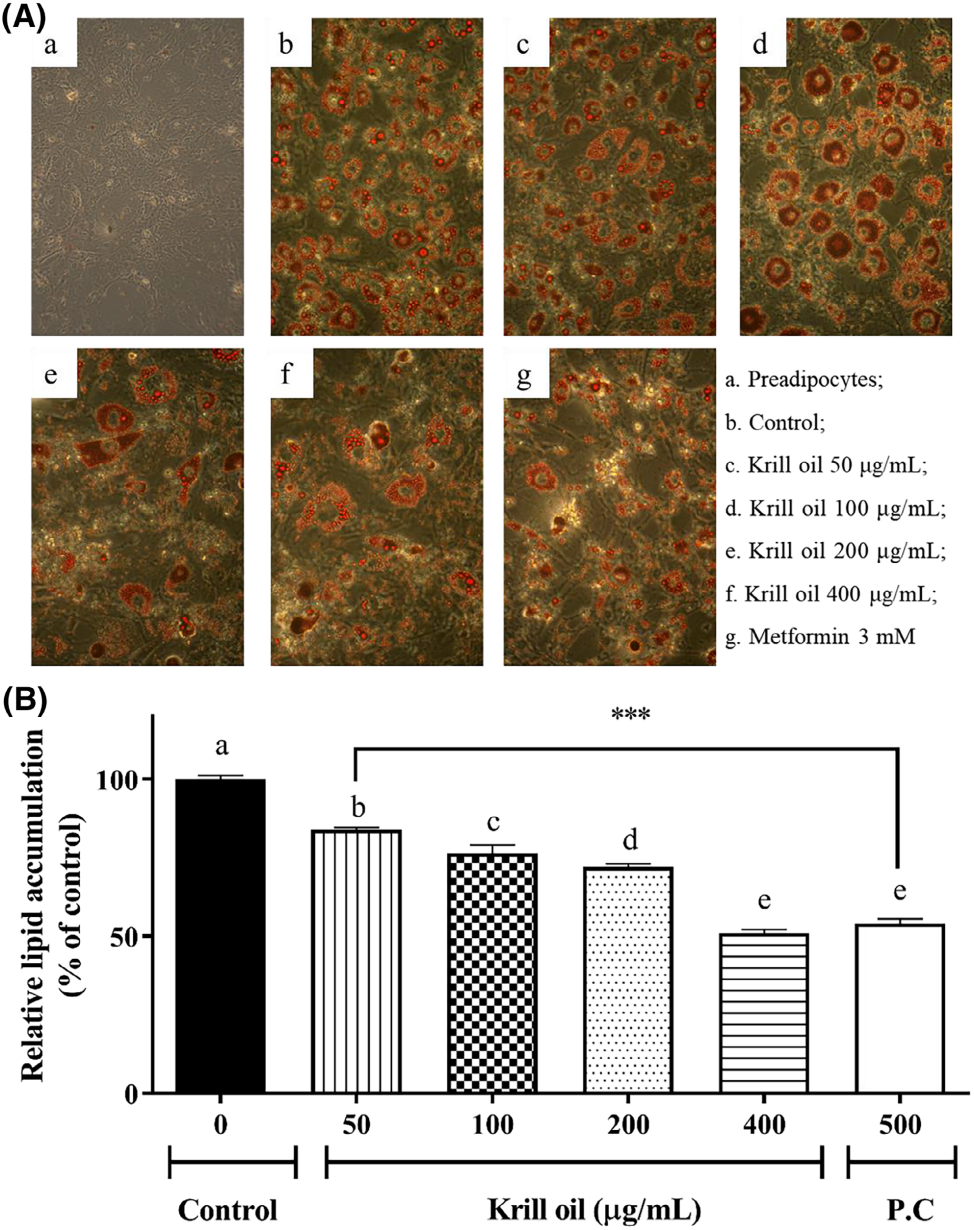
The effects of the krill oil on lipid accumulation. (A) Histology of the typical 3T3‐L1 adipocytes by oil red O staining (original magnification ×200), and (B) the quantification value in the oil red O‐stained 3T3‐L1 cell. The data results are represented as mean ± SD. A value of *p* < .001 versus the control group. P.C, Positive control (metformin).

In order to evaluate the effect of the krill oil on the lipid accumulation, the 3T3‐L1 cells induced fat overload was imaged using cellular lipid droplets that were dyed with a red color using the oil red O method, which is illustrated in Figure 2A, and the contents of the lipid accumulation were then quantitated as a % of the control, which is illustrated in Figure 2B. The adipose differentiation of the 3T3‐L1 cells was confirmed by the form of the oil droplets, which were compared with the preadipocytes group (negative control). The treatment of krill oil seemed to suppress the numbers and sizes of the lipid drop in the differentiated cells (Figure 2A). According to the fixed quantity data obtained from the red dye of the mature 3T3‐L1 cell, the lipid contents in 50, 100, 200, and 400 μg/mL of the krill oil significantly reduced compared to control (*p* < .001). In particular, the 400 μg/mL of the krill oil showed an inhibitory effect on lipid accumulation, and it was similar to metformin 500 μg/mL (positive control).

The study also confirmed that the krill oil reduced the body weight, hepatic lipid droplets, adiposity index, and white adipose mass in high‐fat diet (HFD)‐fed mice. The HepG2 cell, which is like the 3T3‐L1cells, is also able to amass intracellular lipid droplets (Chirambo et al., 2017). Another previous study reported that the treatment of pure phosphatidylcholines reduced fat through the induction of adipocyte cell death in the 3T3‐L1 cells (Kim et al., 2017). The differentiation of adipocytes tends to accumulate the storage of excess lipids and excess triglycerides, so the suppression of lipid production could be one way to prevent obesity. These results imply that krill oil is effective in suppressing the lipid accumulation via oil red O staining. Hence, the current study further investigated the effect of krill oil in regard to regulating the gene expression of the transcription factors that are related to adipogenic differentiation.

### 3.4 Effect of krill oil on the adipogenic gene expression by the protein levels in the 3T3‐L1 adipocyte

**FIGURE 3:**
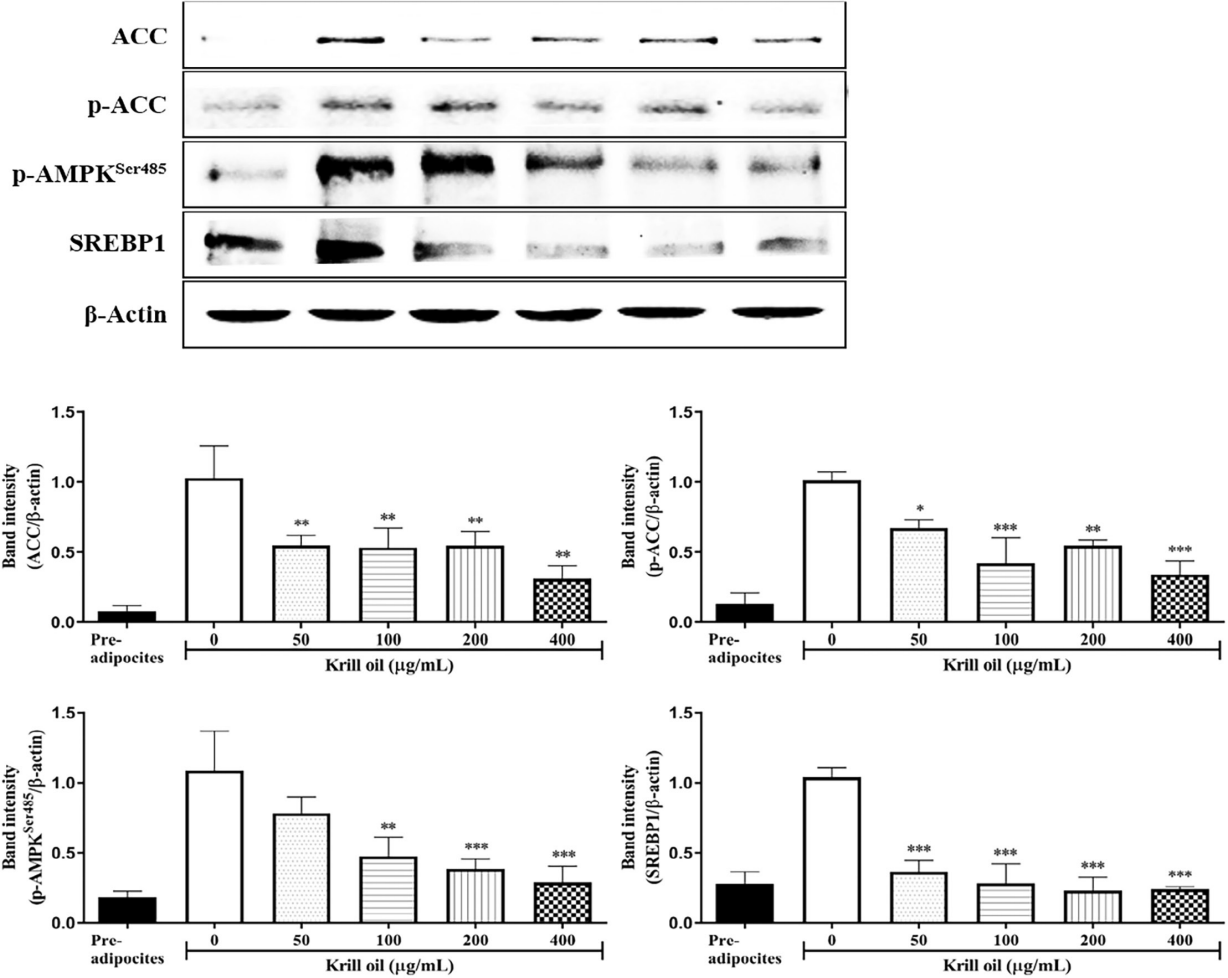
Effects of the krill oil on the expression of adipogenesis markers ACC, p‐ACC, p‐AMPK Ser^485^, and SREBP1 in the 3T3‐L1 adipocytes. Expression levels after differentiation of the 3T3‐L1 cells were determined using a western blot assay (*n* = 3). The data appear as mean ± SD for each of the three experiments. *p < .05, **p < .01, and ***p < .001 as compared with control group, which is the cells that were not treated with krill oil.

**FIGURE 4:**
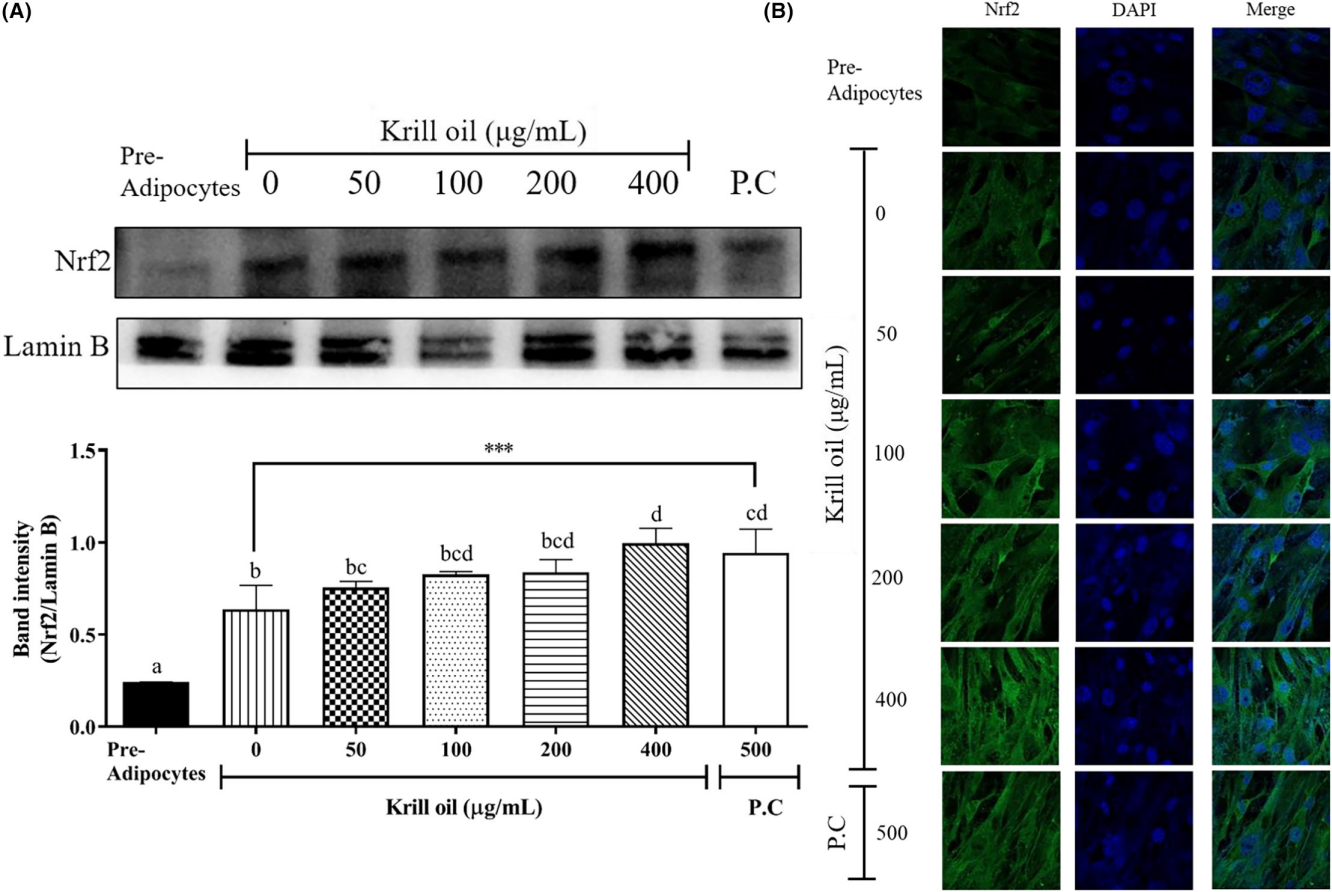
Effects of the krill oil on Nrf2 nuclear levels in 3T3‐L1 adipocytes. (A) Nuclear levels after the differentiation of the 3T3‐L1 cells were assessed using a western blot assay (*n* = 3). The data appear as mean ± SD for each of the three experiments. **p < .01 compared with the control group, which is the cells that were not treated with krill oil. (B) Immunofluorescent staining of Nrf2 after the krill oil treatment. The images were captured using ×400 magnification. P.C, Positive control (metformin).

The 3T3‐L1 adipocytes were exposed to krill oil, and the adipogenic enzymes, which included the p‐AMPK, ACC, p‐ ACC, and SREBP1 expressions were evaluated using the western blot analysis that was described in the Materials and Methods. Previous studies found that mRNA expression of ACC1 and SREBP‐1c was upregulated during adipogenesis in the differentiated 3T3‐L1 cells (Ha et al., 2016; Liu et al., 2019). A comparable finding was observed in the current study that gene expression of adipogenic genes was higher in differentiated 3T3‐L1 adipocyte than preadipocyte. The result is implying that 3T3‐L1 adipocyte cell line model could be useful for investigating the effect of krill on adipogenesis. The phosphorylation of the ACC, which is a lipogenesis relating enzyme, was significantly elevated in the differentiated 3T3‐L1 adipocyte as well as the SREBP1 expression. The AMPK is a sensor protein of cellular energy, which is responsible for adipogenesis (Lee et al., 2011). The AMPK phosphorylation was increased by differentiation, which is shown in Figure 3. The phosphorylation of the ACC and AMPK were attenuated by the krill oil treatment in a dose‐dependent manner, which is illustrated in Figure 4. In addition, the expression of SREBP1 was also reduced by the krill oil treatment. The krill oil inhibited the adipocyte adipogenesis by suppressing the related transcription factor, which is SREBP1, that resulted from the reduction of the AMPK and ACC.

This study evaluated the Ser485 phosphorylation of AMPK, which is shown in Figure 3. The hyper‐phosphorylation of the AMPK at Ser^485^ was detected as differentiated 3T3‐L1 adipocyte, and the krill oil diminished it in a dose‐dependent manner. The activity of the AMPK is regulated by several upstream kinases, such as protein kinase B (PKB) or liver kinase B1 (LKB1) by phosphorylation. It has been published that PKB induces the phosphorylation of the Ser485/491 sites of the AMPK, which subsequently suppresses the Thr172 site phosphorylation of the AMPK that is induced by LKB1 (Horman et al., 2006). The Thr172 site phosphorylation leads to the AMPK activation, so the Ser485 phosphorylation of the AMPK can be regarded as an inhibition mechanism of the AMPK activity (Derkach et al., 2019; Horman et al., 2006). This result implies that the krill oil induces the AMPK activity in the 3T3‐L1 adipocyte. Another study found that the krill oil supplement significantly reduced the triacylglycerol levels and hepatic steatosis with the downregulating of the gene expression, such as the ACC and SREBP‐1c in HFD‐mice (Tandy et al., 2009). Our findings reported that the ACC and SREBP1 were also simultaneously ameliorated by the krill oil treatment, indicating that an application of the krill oil during differentiation process in the 3T3‐L1 adipocyte cell could control promoting adipocytes.

### 3.5 Effect of the krill oil on the Nrf2 transferase activation

In order to confirm whether the krill oil modulated ACC, SREBP1, and AMPK, which resulted from the Nrf2 gene translocation to the nuclear, the nuclear Nrf2 protein level was detected. The differentiated 3T3‐L1 adipocyte was revealed to be higher in the nuclear Nrf2 expression than in the preadipocyte, which is shown in Figure 4A and is similar to another study (D'Archivio et al., 2008). The nuclear Nrf2 level from the cells that were treated with krill oil (400 μg/mL) and the positive control (metformin, 500 μg/mL) were significantly upregulated (*p* < .001) as 1.6‐ and 1.5‐fold compared to the control, respectively (Figure 4A). Also, the upregulated expression of the Nrf2 that was induced by the krill oil treatment was confirmed by the higher intensity of the Nrf2 signal on the immunofluorescence images compared to control, which is illustrated in Figure 4B. Similar to our finding, previous studies reported that a krill oil (40 mg/kg) treatment upregulated the Nrf2 in rats as well as increased Nrf2 activity, contributing to AMPK phosphorylation and the AMPK inhibits reactive oxidative stress and modulates fatty acid biosynthesis (Chang et al., 2018; Helal & El‐Kashef, 2020). In the present study, the nuclear Nrf2 expression and the AMPK activity that was induced by krill oil were significantly upregulated in the 3T3‐L1 adipocytes, whereas the ACC and SREBP1 were downregulated. These results indicated that the adipogenesis inhibition effect of krill oil could be mediated by the Nrf2/AMPK pathway.

## 4 CONCLUSIONS

The current study investigated the molecular mechanisms of krill oil on the antiadipogenic effect that was related to the Nrf2 pathway by measuring the lipid accumulation and the adipogenic key protein genes, such as p‐AMPK Ser^485^, ACC, p‐ACC, and SREBP1. The krill oil was standardized, which contained 50.80% phospholipids, 5.27% myristic acid, and 1.63% linoleic acid. It was acceptable criteria as a food standard for krill oil, which was defined by MFDS. The results from a current study confirmed the inhibition of the lipid accumulation by conducting oil red O staining in the 3T3‐L1 cells treated with krill oil. The ACC, which are the adipogenic enzymes, and SREBP1, which is the adipogenic‐transcription factor, were also simultaneously ameliorated by the krill oil treatment. Furthermore, the krill oil increased the Nrf2 levels, which can induce AMPK activity. It is concluded that krill oil possesses antiobesity activity and inhibition of lipid accumulation, which is mediated by the Nrf2/AMPK pathway in 3T3‐L1 adipocyte.

## AUTHOR CONTRIBUTIONS

**Hyun Jeong Lee:** Conceptualization (equal); formal analysis (lead); software (lead); visualization (lead); writing – original draft (lead). **Ji‐Won Seo:** Formal analysis (equal); methodology (equal). **Yoon Seok Chun:** Data curation (equal); funding acquisition (equal); investigation (equal). **Jongkyu Kim:** Resources (equal); validation (equal); visualization (equal). **Tae‐Gyu Lim:** Resources (equal); software (equal); supervision (equal); writing – original draft (equal); writing – review and editing (equal). **Soon‐Mi Shim:** Conceptualization (lead); resources (lead); supervision (lead); writing – review and editing (lead).

## CONFLICT OF INTEREST STATEMENT

The authors have no conflicts of interest to declare.

## Data Availability

The data that support the findings of this study are available from the corresponding author upon reasonable request.
